# Metabolic Modulators in Depression: Emerging Molecular Mechanisms and Therapeutic Opportunities

**DOI:** 10.3390/ijms26178755

**Published:** 2025-09-08

**Authors:** Kinga Dyndał, Patrycja Pańczyszyn-Trzewik, Magdalena Sowa-Kućma

**Affiliations:** 1 Student’s Science Club of Physiology “NEURON”, Faculty of Medicine, Collegium Medicum, University of Rzeszów, Kopisto 2a, 35-315 Rzeszów, Poland; kd121949@stud.ur.edu.pl; 2 Department of Human Physiology, Faculty of Medicine, Collegium Medicum, University of Rzeszów, Al. Tadeusza Rejtana 16C, 35-959 Rzeszów, Poland; ppanczyszyn@ur.edu.pl; 3 Centre for Innovative Research in Medical and Natural Sciences, Collegium Medicum, University of Rzeszów, Warzywna 1a, 35-310 Rzeszów, Poland

**Keywords:** depressive disorder, GLP-1R agonists, SGLT2 inhibitors, metabolic diseases, PPARα agonists, RAA modulators

## Abstract

Depressive disorder is the most prevalent mental illness, and increasing evidence suggests its potential bidirectional relationship with metabolic disorders. Given the limited efficacy of conventional antidepressants (including Selective Serotonin Reuptake Inhibitors; SSRIs) and the growing prevalence of treatment-resistant depression, there is a significant need to identify alternative molecular pathways underlying the pathophysiology of depressive disorder, which may represent novel therapeutic targets for other agents. Emerging evidence indicates that metabolic dysfunction and depressive disorder share a common pathophysiological molecular mechanism and increase each other’s risk. Targeting peripheral metabolic pathways and their interactions with the central nervous system may alleviate depressive symptoms. Glucagon-Like Peptide-1 agonists (GLP-1 RAs) and Sodium–Glucose Cotransporter-2 (SGLT2) inhibitors, widely used in the treatment of type 2 diabetes and obesity, exhibit neurotrophic and anti-inflammatory effects, ameliorate oxidative stress, and enhance mitochondrial function, collectively contributing to the antidepressant-like effects observed in preclinical studies. Peroxisome Proliferator-Activated Receptor (PPAR) α agonists primarily regulate lipid and glucose metabolism, which may potentially improve neuronal plasticity and mood regulation. Moreover, agents such as Angiotensin Receptor Blockers (ARBs) and Angiotensin Receptor-Neprilysin Inhibitors (ARNIs), used in hypertension treatment, exert central anti-inflammatory and neuroprotective effects via the modulation of the renin–angiotensin–aldosterone system (RAAS), implicated in affective disorders. Nevertheless, long-term, head-to-head trials are required to establish their efficacy, safety, and therapeutic positioning within current treatment paradigms. The aim of this review is to summarize current evidence on metabolic modulators as potential antidepressant strategies, focusing on their molecular mechanisms, preclinical and clinical findings, and prospects for integration into future therapies for depression.

## 1. Introduction

Depressive disorder is the most prevalent and clinically diagnosed mental disorder globally, affecting people of all ages, genders, and social backgrounds. It manifests as significant and persistent low mood and the loss of interest or pleasure for a long time [1]. Depressive disorder affects an estimated 280 million individuals, accounting for about 3.8% of the general population, with a prevalence of approximately 5% among adults [2]. The consequences of depressive disorder may be long-lasting or recurrent, and may substantially affect an individual’s ability to function, both in the family and in society. Additionally, depressive disorders are associated with an increased risk of suicidal thoughts, often leading to suicide attempts and suicide (according to the World Health Organization, over 700,000 people worldwide commit suicide each year) [2]. The underlying pathophysiology of depressive disorder remains incompletely understood. It is widely recognized as a multifactorial disorder that results from the interplay of biological, genetic, environmental, and psychosocial factors [3].

Metabolic disorders are conditions that affect and disrupt any aspect of normal metabolism. The occurrence of metabolic diseases such as type 2 diabetes mellitus (T2DM), obesity, dyslipidemia, and metabolic syndrome poses a severe burden on human society, due to their high morbidity and mortality [4]. While genetics influence the development of metabolic diseases, environmental and behavioral factors also contribute significantly. Research suggests that various behavioral factors, including dietary choices, physical activity levels, and sleep patterns, play a crucial role in influencing the risk and progression of metabolic diseases [5]. Moreover, nutritional components and habits lead to changes in the gut microbiota. Therefore, alterations in the composition of the gut microbiota and the production of microbial metabolites can impact the host’s metabolism and the development of metabolic diseases [6].

The key issue is that metabolic disorders are closely linked to the consequences of depressive disorders. Numerous studies have demonstrated a bidirectional relationship between metabolic diseases and depressive disorders, suggesting shared pathophysiological mechanisms and an increased mutual risk (for review see [7]). Individuals with T2DM have a higher risk of developing depressive symptoms, along with elevated glycemic markers such as glycated hemoglobin (HbA1c), fasting blood glucose (FBG), and postprandial blood glucose (PBG) [8]. Conversely, depressive disorder may contribute to the development of diabetes, potentially through an increased incidence of central obesity [9]. Furthermore, streptozotocin (STZ)-induced diabetic models in mice and rats have been shown to exhibit depressive-like behavior [10,11]. Obesity and depressive disorder may also have a complex bidirectional relationship underpinned by overlapping biological and psychosocial pathways [12]. Dyslipidemia and depressive disorder are also linked, with research suggesting a bidirectional relationship where each condition may influence the other. Patients with depressive symptoms exhibit adverse plasma lipid patterns, characterized by higher levels of triglyceride-glucose (TyG) and lower levels of high-density lipoprotein cholesterol (HDL-C) [13].

Moreover, patients suffering from major depressive disorder (MDD) have been observed to exhibit significantly elevated triglycerides (TG) levels, which are also positively correlated with disease severity [14]. Studies using various animal models, including Flinders Sensitive Line rats (a genetic model of depression), low-density lipoprotein receptor knockout (LDLR−/−) mice (a model of hypercholesterolemia and atherosclerosis) or mice fed a high-fat diet (HFD; a diet-induced model of obesity and metabolic syndrome) have demonstrated a correlation between high cholesterol levels and behavioral symptoms, resembling anxiety-like behaviors and depressive disorders [15,16,17]. Hypertension (HTN) is a common condition in the general population and a major component of metabolic syndrome. There is a reciprocal relationship between emotional state and physical health disorders such as HTN [18]. Emotional and psychosocial stress may play a significant role in the development of HTN, with anxiety and depressive disorder negatively affecting blood pressure regulation. Conversely, patients diagnosed with HTN often experience emotional deterioration following their diagnosis [19].

Our current understanding of the mechanisms underlying depressive disorder is primarily based on the monoamine theory; therefore, selective serotonin reuptake inhibitors (SSRIs), which increase serotonin (5-HT) availability, are the primary treatment for depressive disorder [20]. Unfortunately, this therapy has several notable limitations, such as partial (approximately 30–40% of patients have no complete response) or non-response, delayed onset of action, a risk of relapse after discontinuation, common adverse effects, an increased risk of suicidal thoughts and behaviors during the early stages of treatment, and limited effectiveness in comorbid conditions (e.g., metabolic, inflammatory) [21]. However, depressive disorder is increasingly recognized as a multifactorial disorder that, beyond neurotransmitter dysregulation, is also influenced by long-term stress, altered cytokine signaling, the dysregulation of the hypothalamic–pituitary–adrenal (HPA) axis, mitochondrial dysfunction, oxidative stress, neuroinflammation and impaired neuroplasticity [22,23]. These molecular mechanisms also underline the link between metabolic dysfunction and depressive disorder. Recent advances in metabolic drugs [e.g., Glucagon-Like Peptide-1 (GLP-1) agonists; Sodium–Glucose Cotransporter 2 (SGLT2) Inhibitors; Peroxisome Proliferator-Activated Receptor (PPAR) α agonists; Angiotensin Receptor Blockers (ARBs); Angiotensin Receptor–Neprilysin Inhibitors (ARNIs)] suggest their potential antidepressant effects through the modulation of these pathways [24,25].

Given the limited efficacy of standard pharmacotherapy, the notable relationship between metabolic diseases and depressive disorder, and the multifactorial nature of these conditions, a comprehensive review of metabolic drugs as potential antidepressant agents is both timely and clinically significant. This review aimed to discuss the molecular mechanisms, clinical efficacy, and therapeutic potential of metabolic drugs in the treatment of depressive disorders. A structured literature search was conducted in PubMed/MEDLINE, Google Scholar, Scopus, Embase, and Web of Science databases using the following search terms: ‘depressive disorder’, ‘GLP-1 agonists’, ‘SGLT2 inhibitors’, ‘PPARα agonists’, ‘ARBs’, ‘ARNIs’, and ‘metabolic diseases’. In this review, we summarize recent developments, particularly between 2020 and 2025, in key areas of metabolic agents and their impact on depressive-like behaviors. We examine peer-reviewed clinical and preclinical studies involving animal models of depression, including original research and systematic reviews addressing the effects of these agents on depressive symptoms and the underlying neurobiological mechanisms of depressive disorders. Case reports and non-peer-reviewed sources were excluded from the analysis.

## 2. GLP-1 Receptor Agonists: Antidepressant Effect Beyond Weight Loss 

A substantial amount of empirical research has established a two-way relationship between metabolic disorders and depressive disorder, suggesting shared underlying mechanisms and a mutually reinforcing increase in risk. People diagnosed with T2DM show a higher likelihood of experiencing depressive symptoms, often with elevated glycemic levels. Conversely, depressive disorder may increase the risk of developing T2DM, possibly through mechanisms involving increased central adiposity [26].

Our current understanding of depressive disorder primarily revolves around receptor theory; consequently, SSRIs, which increase the availability of 5-HT, are the primary treatment for depressive disorder in patients with DM [27]. The underlying connection between DM and depressive disorder is complex, influenced by factors such as insulin resistance, oxidative stress, inflammation, HPA axis dysregulation, nervous system dysfunction, and alterations in neurotransmitter systems, including 5-HT and dopamine (DA) pathways [28]. Dysregulation of the HPA axis disrupts neurogenesis in the hippocampus (HP)—a brain region critically involved in the pathophysiology of both T2DM and depressive disorder, due to elevated cortisol levels [29]. Neuroimaging studies indicate that impaired hippocampal neurogenesis contributes to volumetric reductions in this region among individuals with T2DM, mirroring the structural changes observed in individuals with depressive disorder [30]. Diabetes is also associated with significant neuronal loss in key cognitive brain regions such as the frontal, cingulate, and temporal lobes [31]. Furthermore, chronic stress induced by T2DM increases the levels of pro-inflammatory cytokines, which may exacerbate neuroinflammation and depressive disorder via oxidative stress, mitochondrial dysfunction and microglial activation [29].

Higher insulin resistance in T2DM patients correlates with depressive symptoms, and elevated systemic immune–inflammation indices are observed in those with both conditions [32]. Depressive disorder in diabetic patients is linked to poor glycemic control, obesity, and impaired executive function [33]. These multifaceted pathologies limit the efficacy of SSRIs; while they can alleviate depressive symptoms, they do not improve glucose regulation. For example, sertraline reduces depressive symptoms without improving blood glucose, prompting the exploration of treatments with dual antidiabetic and antidepressant effects [34]. Obesity and depressive disorder may have a complex bidirectional relationship underpinned by overlapping biological and psychosocial pathways [35]. Adipose tissue inflammation induces dysregulated leptin secretion and an increased production of pro-inflammatory cytokines, which negatively affect the neurotransmitter systems involved in mood regulation, potentially contributing to depressive symptoms [36]. In individuals with glucose intolerance or chronic hyperglycemia, HPA axis activity is frequently upregulated [29]. Elevated ACTH concentrations with a concomitantly high cortisol concentration reflect a disrupted negative feedback mechanism; hyperinsulinemia further amplifies this hyperactivity, resulting in a dysregulated stress response system linked to depression [30]. Chronic hyperglycemia in T2DM is associated with an increased risk of developing depressive disorders [8]. On the other hand, depressive disorders are likewise marked by the impaired negative feedback regulation of HPA axis function, reflected by reduced glucocorticoid sensitivity, producing persistently elevated cortisol [3]. Excess cortisol, in turn, often promotes gluconeogenesis and worsens the hyperglycemia, reinforcing the bidirectional diabetes–depression relationship [30]. Chronic stress is the most prevalent disorder associated with depressive disorder. High levels of cortisol, persisting for a prolonged period, may lead to the accumulation of visceral fat due to disturbances in carbohydrate metabolism and insulin resistance [12]. Moreover, stress elevates appetite and exacerbates unhealthy eating patterns, thereby contributing to obesity development [37]. Sleep disorders are also associated with depressive disorder. Sympathetic activity increases as a result of a shorter sleep duration, which in turn reduces leptin secretion. The resulting constant feeling of hunger can lead to weight gain. Insufficient sleep is associated with increased ghrelin secretion, a hormone physiologically released to stimulate appetite and food intake. Sleep deprivation may overactivate the HPA axis and increase the secretion of glucocorticoids, leading to disturbed carbohydrate metabolism and insulin resistance. This, in turn, contributes to the development of depressive disorder and obesity [12].

### 2.1. Antidepressant Effects of GLP-1 Receptor Agonists: Evidence from Animal Models

GLP-1 is an incretin hormone primarily secreted by intestinal L-cells in response to nutrient intake, playing a pivotal role in maintaining postprandial glycemic control. GLP-1 receptor agonists (GLP-1 RAs) are antidiabetic agents widely used in the treatment of diabetes, particularly T2DM. By enhancing insulin secretion and suppressing glucagon release, GLP-1 RAs contribute to improved glycemic regulation in patients with T2DM [38]. Moreover, GLP-1 RAs slow gastric emptying by activating GLP-1 receptors located in the gastrointestinal tract. Their central actions provide additional benefits in obesity management, including reductions in body weight, appetite, food cravings, and total energy intake, along with enhanced satiety and the improved regulation of eating behavior [39]. The protective effects of GLP-1 RAs have also been demonstrated in both preclinical and clinical studies, highlighting potential cardiovascular and renal benefits in animal models, as well as in humans [38].

**Figure 1:**
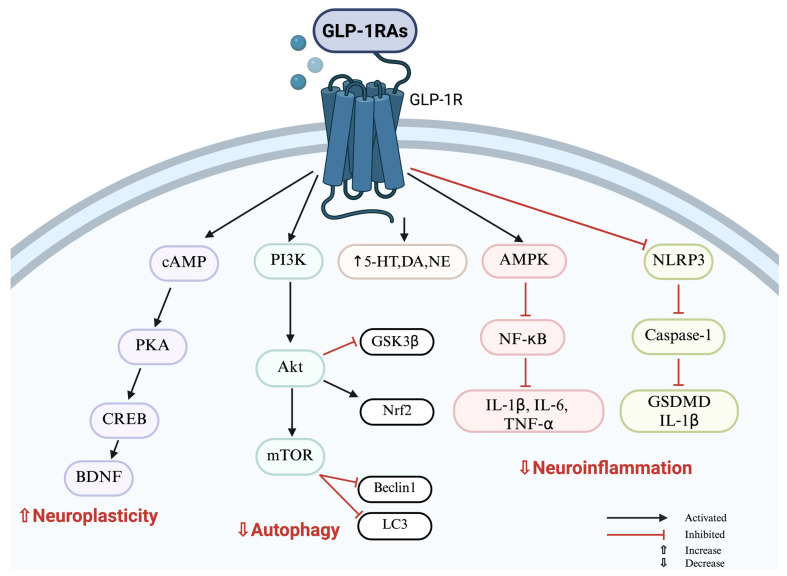
GLP-1 receptor agonists attenuate depressive symptoms through multiple molecular pathways. They increase levels of 5-HT, DA and NE, thereby restoring monoaminergic balance. Activation of the cAMP–PKA–CREB–BDNF cascade promotes neurogenesis. Activation of the PI3K–Akt–mTOR pathway, together with the inhibition of Beclin-1, LC3, and GSK3β, as well as the activation of Nrf2, reduces excessive autophagy. Activation of the cAMP–PKA/Epac (exchange protein directly activated by cAMP) cascade stimulates upstream kinases such as LKB1 (liver kinase B1) and CaMKKβ (Ca²⁺/calmodulin-dependent protein kinase kinase beta), which in turn phosphorylate AMPK at Thr172, leading to its activation. Activated AMPK suppresses NF-κB signaling and the release of pro-inflammatory cytokines (IL-1β, IL-6, TNF-α), and inhibition of the NLRP3 inflammasome prevents caspase-1 activation, thereby blocking GSDMD cleavage and further release of IL-1β—collectively, these mechanisms converge to reduce neuroinflammation and exert antidepressant effects. Abbreviations: GLP-1R—glucagon-like peptide-1 receptor; 5-HT—serotonin; DA—dopamine; NE—norepinephrine; cAMP—cyclic adenosine monophosphate; PKA—protein kinase A; CREB—cAMP response element-binding protein; BDNF—brain-derived neurotrophic factor; PI3K—phosphoinositide 3-kinase; Akt—protein kinase B; mTOR—mechanistic target of rapamycin; Beclin-1—autophagy-related protein Beclin-1; LC3—microtubule-associated protein light chain 3; GSK3β—glycogen synthase kinase-3 beta; Nrf2—nuclear factor erythroid 2-related factor 2; AMPK—AMP-activated protein kinase; NF-κB—nuclear factor kappa-light-chain-enhancer of activated B cells; IL-1β—interleukin-1 beta; IL-6—interleukin-6; TNF-α—tumor necrosis factor alpha; NLRP3—NOD-like receptor family pyrin domain containing 3; GSDMD—gasdermin D.

GLP-1-secreting cells and their receptors have also been identified in the brain (including in the HP and the dorsal raphe nucleus—the main source of serotonergic neurons in the brain), where they exert neuroprotective effects [40] (Figure 1, schematic of GLP-1R-mediated antidepressant mechanisms). Notably, depressive disorders are associated with decreased levels of 5-HT, its transporter (5-HTT), DA, and norepinephrine (NE), alongside the elevated expression of inflammatory and cell-death markers such as GSDMD, NF-κB p65, Interleukin (IL)-1β, IL-6, and Tumor Necrosis Factor (TNF)-α, which are common targets of antidepressant therapies [41,42,43,44]. Insulin resistance, a key factor in T2DM pathophysiology, can further disrupt 5-HT signaling [45]. In animal models of insulin-resistant T2DM, reduced extracellular 5-HT levels correlate with depressive-like behaviors [46]. Martin et al. demonstrated that insulin directly modulates dorsal raphe 5-HT neurons through insulin receptor signaling, and that insulin resistance impairs this mechanism, resulting in disrupted 5-HT neurotransmission via 5-HT1A receptor-mediated inhibitory feedback [45]. Moreover, insulin resistance can lead to elevated levels of branched-chain amino acids (BCAAs) [47]. Since BCAAs share the L-type amino acid transporter 1 (LAT1) with tryptophan (TRY), at the blood–brain barrier (BBB), higher BCAA concentrations limit TRY transport and reduce 5-HT synthesis [47]. As shown in Figure 1 (schematic of GLP-1R–mediated antidepressant mechanisms) and Table 1 (preclinical studies summary), recent findings suggest that GLP-1 receptor agonists (GLP-1 RAs) possess significant neuroprotective and antidepressant potential, particularly in the hippocampus. They enhance levels of 5-HT, 5-HTT, DA, and NE, supporting monoaminergic balance, while also improving neuronal insulin sensitivity and counteracting central insulin resistance, thereby promoting 5-HT signaling (Table 1). Emerging evidence suggests that mitochondrial dysfunction is a key contributor to the pathogenesis of depressive disorder [3]. Mitochondria play a central role in ATP production, maintaining membrane stability, regulating redox homeostasis, and promoting antiapoptotic signaling, as well as buffering intracellular calcium and influencing the regulation of neuroplasticity and neurotransmission. Disturbances in mitochondrial function lead to the excessive generation of reactive oxygen species (ROS), which in turn promote the release of mitochondrial DNA (mtDNA) and pro-inflammatory mediators [3]. Hyperglycemia further exacerbates these processes by enhancing aerobic glycolysis and overloading the mitochondrial electron transport chain, resulting in electron leakage, superoxide formation, and mitochondrial dysfunction [29]. Mitochondrial impairment may also trigger microglial activation. Released mtDNA and ROS stimulate the NLRP3 inflammasome, leading to caspase-1 activation and the cleavage of gasdermin D (GSDMD), thereby inducing pyroptosis. Caspase-1 additionally activates IL-1β and IL-18, amplifying the inflammatory cascade [48]. Activated microglia release pro-inflammatory cytokines, contributing to neuroinflammation and oxidative stress—processes consistently observed in depressive disorder [49]. GLP-1 RAs have been shown to counteract these mechanisms by reducing neuroinflammation and microglial pyroptosis through the downregulation of GSDMD and IL-1β (Figure 1, schematic of GLP-1R-mediated antidepressant mechanisms), (Table 1, preclinical studies summary). They also decrease the expression of ionized calcium-binding adapter molecule 1 (Iba1), a marker of microglial activation [50], and lower levels of pro-inflammatory cytokines such as IL-6 and TNF-α, as well as NF-κB p65. Moreover, GLP-1 RAs mitigate apoptosis (via downregulation of BAX), necrocytosis, and oxidative stress (e.g., TBARS, GSH, SOD) (Table 1). Impaired AMPK (AMP-activated protein kinase) phosphorylation and inactivation have been linked to depression-like behaviors [51]. GLP-1 RAs may reverse these processes by inducing the cAMP–PKA/Epac signaling cascade, which subsequently activates upstream kinases such as LKB1 and CaMKKβ, leading to the phosphorylation of AMPK at Thr172 [52] (see Figure 1, schematic of GLP-1R-mediated antidepressant mechanisms and Table 1, summary of preclinical studies). Thus, activating the AMPK signaling pathway has been associated with a reduction in depressive-like behaviors [51]. Finally, alterations in autophagy signaling have also been observed in individuals with depressive disorder [53].

GLP-1 RAs reduce autophagy by decreasing Beclin-1 and LC3 expression. Liraglutide, in particular, activates Nrf2 via the PI3K/Akt pathway and inhibits HMGB1 expression, protecting against corticosterone-induced hippocampal damage and enhancing GSK3β phosphorylation (Table 1, preclinical studies summary). Nrf2 plays a key role in depressive disorder by regulating the cellular antioxidant response. Notably, studies indicate that the Klotho protein plays a key role in the pathophysiology of depressive disorder, particularly through the modulation of oxidative stress, inflammation, and glutamatergic neurotransmission by enhancing Nrf2 activity [54]. Furthermore, differences in Klotho levels may affect the effectiveness of antidepressant therapy, and its effects appear to be dependent on age, gender and genetic factors [55]. To date, no studies have specifically investigated the effects of GLP-1 analogs on the Klotho/Nrf2 signaling pathway. It highlights a promising avenue for future research.

Hippocampal neurogenesis is impaired in adults with depressive disorder [56]. Doublecortin (DCX) is a marker of immature developing neurons and an essential factor in neurogenesis. In animal models of depression, especially following chronic unpredictable mild stress (CUMS), DCX levels in the HP and olfactory bulb are significantly decreased, indicating impaired neurogenesis, which correlates with the development of depressive-like behaviors in rodents [57,58]. cAMP response element binding protein (CREB) plays a key role in signaling to regulate adult neurogenesis. CREB expression is significantly decreased in patients with depressive disorder [59]. Synaptophysin (SYN) is the major integral membrane protein of synaptic vesicles. In depressive disorder, SYN levels are consistently reduced in the HP and cortex, reflecting synaptic loss and impaired neuroplasticity [60]. Deficiencies or imbalances in BDNF lead to malfunction of synaptic plasticity and contribute to the development of depressive disorder [61]. Moreover, mitochondrial dysfunction can induce oxidative stress that inhibits the Bcl-2-dependent signaling, reduces CREB transcriptional activity, and downregulates BDNF expression [48]. Additionally, increased ROS causes cell membrane lipid peroxidation, which may cause severe membrane damage and potential cell death. Mitochondrial dysfunction may also lead to mitochondrial depolarization and the inhibition of OXPHOS, open the mitochondrial permeability transition pore, and trigger the activation of cytochrome c–caspase-9/3, ultimately leading to apoptosis [3]. This mitochondria-driven apoptosis contributes to hippocampal cell loss, thereby impairing synaptic plasticity and neurogenesis [29]. Hippocampal neurons are especially vulnerable to oxidative stress, owing to their high oxygen demand, polyunsaturated fatty-acid-rich membranes and comparatively limited antioxidant levels [62]. Furthermore, the HP represents one of the limited neurogenic niches of the adult brain, where newly generated neurons exhibit heightened susceptibility to ROS, thereby exacerbating the region’s oxidative vulnerability [62]. The PI3K/AKT signaling pathway plays a pivotal role in antidepressant effects through its anti-inflammatory properties, the modulation of neurotransmitters, the promotion of neurogenesis, and the enhancement of synaptic plasticity [63]. Diminished cAMP-PKA signaling affects neuronal excitation as well as synaptic plasticity, leading to the pathological manifestation of depressive disorder [64]. GLP-1 RAs may modulate key neuroplasticity and survival pathways by upregulating DCX, p-CREB, SYN, BDNF, PI3K, Akt, p-Akt, and PKA (Table 1). Glycerophospholipids and sphingolipids are key components of neuronal membranes and myelin, as well as main regulators of synaptic function. The disruption of glycerophospholipid and sphingolipid metabolism may cause abnormal cell function in the prefrontal cortex (PFC) and HP regions, leading to depression-like cognitive dysfunction [65]. Dulaglutide modulates key lipids such as sphingolipids, lysophosphatidylcholine (LPC), lysophosphatidylethanolamine (LPE), phosphatidylcholine (PC), phosphatidylethanolamine (PE), and phosphatidylinositol (PI), which are all implicated in the pathological alterations associated with depressive disorder [66,67]. Increased PE and PC levels are closely linked to anxiety and depressive-like behaviors in individuals with MDD. LPC and LPE are significantly increased in MDD and show pronounced positive relationships with depression severity [68]. Dulaglutide, by restoring lipid balance, may potentially exert antidepressant effects [67]. N-acetyl-L-aspartic acid (NAA) is considered a marker of neuronal density and integrity in the brain, representing a primary metabolic pathway across various brain regions. Studies have shown that in rat models of CSDS and chronic unpredictable mild stress (CUMS), NAA levels are reduced in the amygdala (AMY) [69]. Dulaglutide may increase NAA levels in the hippocampus, indicating that it may be a potential therapeutic target for depressive disorder [67]. In the chronic mild stress model of depression (CMS), L-glutamic acid and L-arginine are upregulated. Dulaglutide decreased the levels of L-glutamic acid. Arginine, a precursor of proline, is converted to nitric oxide and citrulline [70]. Dulaglutide decreases arginine and proline and may thereby indirectly reduce NO production, exerting a neuroprotective effect [67]. Furthermore, dulaglutide treatment increased succinic acid, creatine, gentisic acid, ADP, guanosine monophosphate, and adenosine monophosphate [67]. Adenosine and guanosine may modulate cognitive function and are related to anxiety and depressive disorders. Guanosine has been shown to exert antidepressant effects [71]. Postmortem analyses of brain structures from suicide victims showed decreased levels of GFAP mRNA and protein, indicating astrocyte dysfunction [72]. NSE levels are increased in the serum and cerebrospinal fluid of patients with depressive disorder and can directly correlate with neuronal damage [73]. Increased peripheral C-reactive protein (CRP) and galectin-3 (GAL3) levels correlate with neuroinflammatory activity [74].

**Table 1:** Summary of preclinical studies on GLP-1 RAs: effects on depressive-like behaviors and molecular pathways.

*Species/* *Strain/Cell Line*	*Animal Model/* *Treatment*	*Results-Behavioral Tests*	*Samples*	*Methods*	*Results*	*References*
**Male C57BL/6 mice (8–10 weeks old)**	High Fat Diet (HFD; 12 weeks); Semaglutide (0.05 mg/Kg; i.p.)/Fluoxetine (20 mg/Kg; p.o.; 6 weeks; *n* = 15)	↓ Immobility time in the tail suspension test and forced swim test; ↑ The alternation index in T Maze Spontaneous Alternation; ↑ Time spent in the open arm; ↓ time spent in the enclosed arms in elevated plus Maze Test	Hippocampus (HP), distal colon, feces, and serum	ELISA Immunostaining Western blot	↓ Serum IL-1β and LPS ↑ Gut GLP-1 expression ↓ p-NF-κB, TNF-α, IL-6, IL-1β, nitrotyrosine in HP ↑ Brain 5-HT, 5-HTT, NMDAR1, NMDAR2, Glu2R, p-AMPK in HP ↑ GLP-1R expression HP	[52]
**Male db/db mice (5 weeks old)**	Diabetes-induced depression model; Exendin-4 (5 μg/kg; 4 days; i.p.; *n* = 9)	↓ Immobility time in the tail suspension test and forced swim test; ↑ Total distance traveled in the open field test	Hippocampus (HP), Amygdala (AMY), Anterior cingulate cortex (ACC)	ELISA Immunostaining Western blot	↑ Microglial GLP-1R expression HP ↓ Activation of microglia ↓ ASC, caspase-1, IL-1β, GSDMD, ROS	[75]
**Male C57 BL/KsJ db/db mice (16 weeks old)**	Diabetes-induced depression model; Semaglutide (0.05 mg/kg; 8 weeks; s.c.; *n* = 10)	↑ Platform area crossing and time spent in the target quadrant in the Morris water maze test ↑ Total distance in the central zone, wall-climbing counts in the open field test	Hippocampus (HP)	qRT-PCR Western blot HE staining	↑ Neurons in the CA1, CA3, and DG regions in HP ↑ BDNF in HP	[76]
**Mice hippocampal neuronal cell line HT22**	Corticosterone (CORT; 200 μM) + high glucose (HG 50 mM) 48 h; GLP-1 (50 nM)	n.a.	Hippocampus (HP)	Flow Cytometry CLSM ELISA Western blot	↓ Apoptosis and necrocytosis rates, LDH, glucose concentrations ↑ BDNF, 5-HT, DA, NE ↑ PKA, p-CREB, and p-Trkb	[77]
**Male Wistar rats (6 weeks old)**	High Fat Diet (HFD; 12 weeks); Liraglutide (300 µg/kg/day;4 weeks; s.c.; *n* = 12)	↑ Sucrose consumption in the sucrose preference test ↓ Immobility time in the forced swim test ↑ Crossed squares, ↓ time and latency to leave in the central zone in the open field test ↓ Time to reach the platform, ↑ time spent in the target quadrant in the Morris water maze test	Serum Hippocampus (HP)	RT-qPCR ELISA TEM	↑ PI3K, Akt, mTOR, ↓ Beclin 1, LC3 HP gene expression ↑ PI3K, Akt, p-Akt, BDNF, p-mTOR, ↓ Beclin 1, LC3 proteins in HP ↓ Corticosterone in serum; TNF-α, IL-6 in HP	[78]
**Male Wistar rats (2 months old)**	Lipopolysaccharide (LPS; 0.25 mg/kg); Exendin-4 (0.1 μg/kg *n* = 10; 0.3/0.5 μg/kg; i.p.; *n* = 9)	↔ Immobility, swimming, climbing time in the forced swim test ↔ Crossed squares in the open field test	Serum Hippocampus (HP)	ELISA Colorimetric Assay	↓ IL-6, ↑ BDNF in HP ↓ TBARS serum	[79]
**Male Swiss albino mice** **(weighing 25–35 g)**	Dexamethasone-induced depression (32 mcg/kg; 7 days); Liraglutide (200 mcg/kg; 28 days)	↓ Immobility time in the tail suspension test and forced swim test ↑ Crossed squares in the open field test ↑ Sucrose consumption in the sucrose preference test	Hippocampus (HP) Prefrontal cortex (PFC) Brains left halves (BLH)	ELISA	↑ 5-HT, DA, NE in HP, PFC ↓ NLRP, IL-1β; ↑Neuropeptide Y, IL-10 in BLH ↑ CREB, BDNF, PSD95; ↓ NMDAR2 in BLH	[80]
**Male C57BL/6 mice (8–10 weeks old)**	Chronic unpredictable mild stress (CUMS; 4 weeks); Liraglutide (300 μg/kg/day; i.p.; *n* = 10)	↓ Immobility time in the tail suspension test and forced swim test	Serum Hippocampus (HP) Prefrontal cortex (PFC)	ELISA qRT-PCR Western blot	↓ CORT in serum ↑ Nrf2, ↓ HMGB1 in HP and PFC ↓ IL-1β, IL-6, TNF-α; ↑ GSH, SOD in HP and PFC	[81]
**Male C57BL/6N mice (8 weeks old)**	Corticosterone (CORT; 35 μg/mL/d; 30 days); Liraglutide (20 nmol/kg; 15 days; i.p.)	↑ Mobility time in the tail suspension test and forced swim test ↑ Exploration time in the open field test	Plasma Hippocampus (HP)	ELISA Western blot	↓ ACTH in plasma ↑ GSK3β phosphorylation in HP ↑ DCX in HP	[82]
**Male ICR mice (7 weeks old)**	Chronic mild stress (CMS; 4 weeks); Dulaglutide (0.3/0.6 mg/kg; 3 weeks; i.p.; *n* = 13–15)	↓ Immobility time in the tail suspension test and forced swim test	Hippocampus (HP)	LC-MS/MS	Regulation PC, PE, PI, LPC and LPE, ↑ NAA,↓ L-glutamic acid, L-arginine, proline in HP ↑ succinic acid, creatine, gentisic acid, ADP, GMP, AMP	[67]
**Male C57BL/6N mice (8 weeks old)**	Chronic unpredictable mild stress (CUMS; 40 days); Lixisenatide (10/50 nmol/kg/d; i.n.; 25 days)	↓ Immobility time in the tail suspension test and forced swim test ↑ Time spent in center area in open field test ↑ Time spent in open arm in elevated plus maze test	Olfactory bulbs (OB) Hippocampus (HP)	Western blot	↑ CREB phosphorylation level ↑ DCX in HP DG region, OB	[83]
**Male CD-1 mice (7 weeks old)**	Streptozotocin (STZ; 40 mg/kg; 5 days); Semaglutide (0.21/0.42/0.03/0.06 mg/kg; 2 weeks; s.c.; *n* = 8)	↑ Crossed squares, ↑ time spent in the central zone in the open field test ↓ Immobility time in the forced swim test	Perftontal cortex (PFC) Serum	ELISA HPLC	↓ GFAP, NSE, GAL3 in PFC ↓ CRP in serum	[84]
**Male ICR mice (weighing 18–22 g)**	Repeated restraint stress (RRS; 30 days); Geniposide (50/100mg/kg); Fluoxetine (20 mg/kg); 15 days; i.g.; *n* = 12)	↑ Sucrose consumption in the sucrose preference test ↑ Crossed squares in the open field test ↓ Immobility time in the tail suspension test and forced swim test	Hippocampus (HP)	ELISA Immunostaining Western blot	↓ Apoptosis of neurons in HP ↓ cleaved Caspase-3, Bax/Bcl-2 ratio) ↓ IL-1β, TNF-α in HP ↑ GLP-1R expression, p-GSK3β, p-AKT in HP	[85]
**Male Sprague Dawley rats (18–20 months old)**	Surgery-induced trauma Exendin-4 (5 µg/kg/day; 14 days; i.p.; *n* = 30)	↑ Platform area crossing and time spent in the target quadrant in the Morris water maze test ↑ Total distance traveled, time spent in center area in the open field test	Hippocampus (HP)	Western blot Immunostaining	↓ NF-κB p65, IL-1β in HP ↓ Iba-1, ↑ SYN, p-GSK-3β in HP ↑ GLP-1/GLP-1R expression in HP	[86]

Abbreviations: i.p.—intraperitoneally; s.c.—subcutaneously; i.n.—intranasally; i.g.—intragastrically; p.o.—per-orally; (↑)—increase while; (↓)—decrease; (↔)—no changes; n.a.—not applicable

### 2.2. Antidepressant Effects of GLP-1 Receptor Agonists: Evidence from Human Studies

Emerging evidence suggests that GLP-1 receptor agonists also exert antidepressant and anxiolytic effects in humans [24,87,88,89]. A nationwide cohort study from Taiwan (2022) demonstrated a lower cumulative incidence of anxiety and/or depression among GLP-1-RA users compared to non-users (6.80 vs. 9.36 per 1000 person-years). Importantly, the treatment effect was significant in women [24]. Furthermore, a multicenter cohort study conducted between 2019 and 2022 showed that 12 months of GLP-1RA treatment was associated with significant reductions in outpatient hospital visits for depression and office visits for both depression and anxiety compared with DPP-4i use. Additionally, these beneficial effects were most evident for semaglutide, liraglutide, and dulaglutide [87]. Another retrospective cohort study also suggested an advantage of GLP-1RA treatment compared to DPP-4i for suicide attempts. Patients with T2DM treated with GLP-1RA exhibited a consistently lower risk of suicide attempts compared with those receiving DPP-4i (with an OR 0.461 (95% CI: 0.366–0.58), *p* <  0.001), with a significant effect observed among individuals with a history of depression or prior suicide attempts (OR 0.377 (95% CI: 0.285-0.499), *p*  <  0.001) [88]. In line with these findings, Wium-Andersen et al. reported that low doses of metformin, DPP4 inhibitors, GLP1 agonists, and SGLT2 inhibitors were associated with a reduced risk of depressive disorder in patients with diabetes compared with non-users, with the greatest risk reduction observed for SGLT2 inhibitors (OR 0.55, 95% CI: 0.44–0.70). Conversely, the use of insulin, sulfonylurea, and high-dose metformin was associated with an increased risk of depression [89]. However, growing evidence also suggests a potentially opposite role of GLP-1RA in the modulation of depression-related behaviors [90,91,92,93,94,95,96,97]. A disproportionality analysis of adverse event databases from the US (FAERS), Canada (CVAROD) and Australia (DAEN) (2025) revealed significant associations between semaglutide and depressive symptoms (FAERS, ROR = 6.24, CI: 4.49–8.69), panic attacks (FAERS, ROR = 1.46, CI: 1.16–1.82) and suicidal ideation (FAERS, ROR = 2.58, CI 2.31–2.88). Likewise, liraglutide was linked to depression (CVAROD, ROR = 1.68, CI: 1.12–2.51), whereas dulaglutide demonstrated significant associations with eating disorders (FAERS, ROR = 1.47, CI: 1.26–1.71) and insomnia (FAERS, ROR = 2.93, CI: 2.35–3.66) [98]. Similar findings were reported in a large community-based cohort study, which demonstrated a significant association between GLP-1 RA treatment and an overall 98% increased risk of any psychiatric disorders. Notably, patients receiving GLP-1 RAs exhibited a 108% increased risk of anxiety, a 195% higher risk of major depression and a 106% elevated risk of suicidal behavior. These associations were primarily observed with liraglutide and semaglutide [90]. An analysis of the EudraVigilance database (2021–2023) identified 31,444 adverse event reports related to semaglutide, liraglutide and tirzepatide, of which 372 (1.18%) involved psychiatric adverse events, with women accounting for 65% of these reports. The most frequently reported events were depressive disorder (50.3%), anxiety (38.7%) and suicidal ideation (19.6%). Notably, nine fatal cases, predominantly among men (8 out of 9), were related to completed suicides and depression) and 11 life-threatening outcomes were observed, primarily associated with liraglutide and semaglutide [91]. A VigiBase analysis demonstrated similar findings, showing that among 2,061,901 reports, 21,414 involved psychiatric adverse drug reactions with GLP-1RAs, with semaglutide demonstrating significant disproportionality signals for anxiety (aROR: 1.26, 95% CI: 1.18–1.35), depressive disorders (aROR: 1.70, 95% CI: 1.57–1.84) and suicidality (aROR 1.45, 95% CI: 1.29–1.63) [92]. A retrospective pharmacovigilance study analyzing FAERS data (2004–2024) demonstrated that tirzepatide was associated with significantly lower mortality rates (0.26%) compared with those of other GLP-1RAs, including liraglutide and semaglutide [93]. Additionally, three clinical cases reported the onset of depressive symptoms following semaglutide treatment initiation. One case involved a man in his late 70s with T2DM and no prior psychiatric history who attempted suicide approximately one month after starting semaglutide treatment [94]. The other two cases described middle-aged patients: a 54-year-old man with no previous history of depression and a 40-year-old female patient with recurrent major depressive disorder [95]. Both patients developed or experienced a recurrence of depressive symptoms within a month of semaglutide use. In all three cases, psychiatric symptoms improved or resolved after semaglutide discontinuation, suggesting a potential association between the drug and depression or suicidality. Furthermore, in a cross-sectional study of 43 patients with T2DM, exenatide users demonstrated higher Patient Health Questionnaire-9 (PHQ-9) scores and Perceived Stress Scale (PSS) scores compared with non-users [96]. Although psychiatric adverse events represented only a minor fraction of all adverse reports associated with GLP-1RAs, their clinical severity and fatal outcomes highlight the need for further investigation. Moreover, the inconsistency of safety data regarding GLP-1RA due to their heterogeneous effects on depressive symptomatology and suicidal behavior underscores the complex and multifactorial pathogenesis of depressive disorder, especially in patients with T2DM. Conflicting findings may arise from study design (e.g., observational vs. RCTs) or patient cohorts (e.g., individuals with T2DM with or without comorbid depression) [24,87,88,89,90,91,92,93,94,95,96]. This highlights the importance of individualized therapeutic approaches, rigorous psychiatric monitoring during GLP-1RA treatment, and well-designed prospective studies to evaluate their efficacy and neuropsychiatric safety profile comprehensively.

## 3. Sodium-Glucose Cotransporter 2 (SGLT2) Inhibitors and Their Emerging Role in Mood Regulation

Sodium–glucose co-transporter-2 (SGLT2) inhibitors are a novel class of oral antidiabetic drugs that represent a significant advancement in the management of type 2 diabetes mellitus. These drugs target the SGLT-2 proteins expressed in the proximal convoluted tubules. SGLT2 inhibitors suppress the reabsorption of glucose from the tubular lumen by inhibiting SGLT2, thereby increasing urinary glucose excretion and ameliorating hyperglycemia [97]. Furthermore, they reduce body weight and visceral fat, while improving multiple metabolic disturbances associated with metabolic syndrome, including blood pressure, lipid profile, and serum uric acid levels [99].

**Figure 2:**
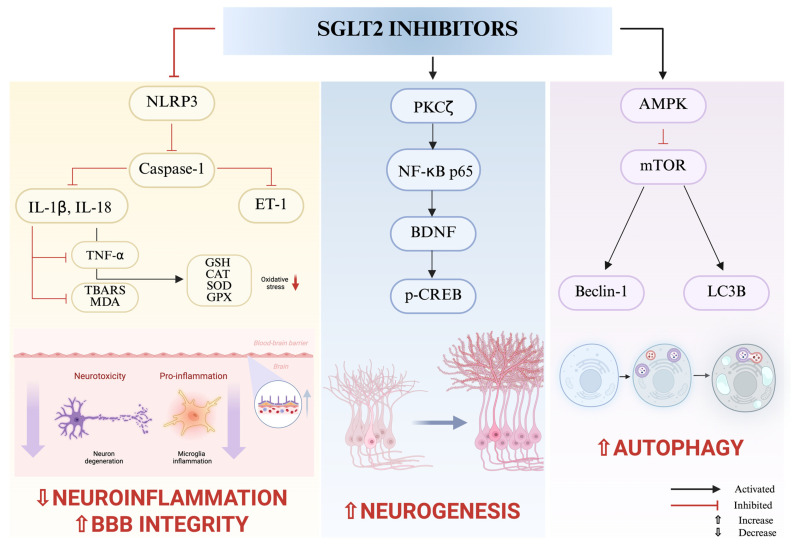
SGLT2 inhibitors act on multiple molecular targets to reduce depressive symptoms. SGLT2 inhibitors attenuate depressive symptoms through multiple molecular mechanisms. Inhibition of the NLRP3 inflammasome suppresses caspase-1 activation, thereby reducing ET-1 and IL-1β, IL-18, which in turn decreases TNF-α, TBARS, and MDA levels. Lower concentrations of IL-1β enhance antioxidant enzymatic systems, including GSH, CAT, SOD, and GPX, ultimately reducing oxidative stress. These effects converge to attenuate neuroinflammation and increase BBB integrity. Activation of the PKCζ–NF-κB p65–BDNF–p-CREB signaling pathway promotes neurogenesis. Furthermore, activation of AMPK inhibits mTOR and stimulates Beclin-1 and LC3B, thereby enhancing autophagy. Collectively, these mechanisms contribute to neuroprotection and antidepressant effects. Abbreviations: SGLT2—sodium-glucose cotransporter 2; NLRP3—NOD-like receptor family pyrin domain containing 3; ET-1—endothelin-1; IL-1β—interleukin-1 beta; IL-18—interleukin-18; TNF-α—tumor necrosis factor alpha; TBARS—thiobarbituric acid reactive substances; MDA—malondialdehyde; GSH—glutathione; CAT—catalase; SOD—superoxide dismutase; GPX—glutathione peroxidase; PKCζ—protein kinase C zeta; NF-κB p65—nuclear factor kappa-light-chain-enhancer of activated B cells p65 subunit; BDNF—brain-derived neurotrophic factor; p-CREB—phosphorylated cAMP response element-binding protein; AMPK—AMP-activated protein kinase; mTOR—mechanistic target of rapamycin; LC3B—microtubule-associated protein 1 light chain 3 beta.

Recent studies have shown their beneficial impact on the brain [100]. SGLT2 expression has been identified in various brain regions, including HP, AMY, the hypothalamus, periaqueductal gray (PAG), endothelial cells in the BBB, lateral habenula (LHb) and in the dorsomedial medulla–nucleus of the solitary tract (NTS) [101]. The HP, AMY, hypothalamus, LHb and NTS play a role as the brain regions implicated in depressive disorder [102,103]. Beyond their role in glycemic control, emerging evidence also suggests the potential of SGLT2 inhibitors as neuroprotective agents, highlighting their antioxidant, anti-inflammatory, and anti-apoptotic effects, as well as their ability to promote angiogenesis and neurogenesis (Figure 2, schematic of SGLT2–mediated antidepressant mechanisms), and (Table 2, summary of preclinical studies).

The LHb, an essential nucleus in the brain, plays a role in multiple behavioral functions such as cognitive and reward processing, mood regulation and motivation. The pathological dysfunction of LHb may impair neurotransmitter signaling, including 5-HT and DA, thereby inducing negative affect and helplessness. Additionally, hyperactivity in the LHb may contribute to dysphoric states and reward avoidance [103]. Taken together, these features are characteristic of mood disorders, including affective and depressive disorders. Moreover, increased blood glucose has been shown to increase LHb neuronal activity in rats [104]. Thus, DM may induce the occurrence and development of depressive disorder due to increased LHb neuronal activity. Furthermore, deep electrical stimulation of the LHb has been shown to alleviate stress-induced depressive-like behavior, accompanied by AMPK activation in the LHb, which correlates positively with antidepressant efficacy [105].

Local and systemic inflammations pose significant risks to the integrity of the blood–brain barrier (BBB), contributing to its disruption and the consequent infiltration of immune cells, the accumulation of metabolic waste, and the dysregulation of neuronal function [106]. The NOD-, LRR-, and pyrin domain-containing protein 3 (NLRP3) inflammasome is an intracellular multiprotein complex. Disruption of the BBB, neuroinflammation, and impaired neurogenesis may induce depressive-like behaviors by promoting NLRP3 activation. NLRP3 may activate inflammatory pro-caspase-1 and indirectly trigger the release of ET-1, contributing to disturbances in the endothelin (ET) system [107]. In depressive disorder, elevated ET-1 levels and altered endothelin receptor expression—notably increased ETAR and decreased ETBR—have been linked to stress-induced neuroinflammation, impaired neurogenesis, and vascular dysregulation [108].

Autophagy plays a key role in stress-related disorders. Abnormal autophagy is closely linked to the occurrence and development of depressive disorder. Moreover, the regulation of autophagy plays a crucial role in the pathophysiology of depressive disorder. AMPK and mTOR are pivotal regulators of autophagy-related proteins, including microtubule-associated protein light chain 3 (LC3B) and Beclin1. In depressive disorder, aberrant activation of the AMPK and mTOR pathway may contribute to the inhibition of autophagy, mitochondrial dysfunction, and impaired synaptic plasticity [109]. Elevated m-TOR levels may weaken the autophagy signaling parameters (such as AMPK, ATG13, Beclin-1, and LC3-II/I). However, the phosphorylation (AMPK α-subunits at Thr-172) of AMPK may suppress the negative autophagic regulator mTOR, thus enhancing autophagy. The decline in the regulatory protein m-TOR enhances the autophagic signaling parameters, reducing neuroinflammation and improving neuroplasticity [110]. Furthermore, impaired autophagy increases ROS and is linked to NLRP3 inflammasome activation [111]. NF-κB interacts with autophagy in a context-dependent manner, influencing inflammation, neuronal survival, and Beclin1 expression. Phosphorylated NF-κB mediates the secretion of BDNF under the influence of protein kinase C (PKCζ)—which enhances neurogenesis and neuroplasticity [112].

TRY metabolism, especially involving 5-HT and kynurenine (KYN), plays a key role in the development of depressive disorder. Pro-inflammatory cytokines, implicated in the etiology of depressive disorder, correlate with the enhanced activity of the TRY-degrading enzyme indoleamine 2,3 dioxygenase (IDO). Increased IDO activity may subsequently induce TRY depletion—the precursor of 5-HT—and the dysregulation of neurotransmission, as well as alterations in the KYN metabolic pathway [3]. Zong et al. identified that elevated KYN levels are causally linked to a higher risk of developing depressive disorder [113]. Conversely, Chen et al. associated altered TRY/KYN metabolism with the depressive behaviors observed in patients with inflammatory bowel disease (IBD) [114]. IBD is characterized by intestinal inflammation and gut dysbiosis, which involves an imbalance in the composition and function of the gut microbiota and compromised intestinal integrity. These alterations have also been linked to the development of several mental illnesses, including depressive disorder [6]. β-catenin, Claudin-1, E-cadherin and ZO-1 are key proteins involved in maintaining the structural and functional integrity of intercellular junctions, particularly in the gut [115]. Furthermore, the elevation of pro-inflammatory cytokines is associated with a notable increase in markers of astrocyte (GFAP) and microglial (Iba and CD86) activation [116]. Oxidative stress results from the imbalance between the production of reactive oxygen species and the body’s antioxidant mechanisms, and is associated with depressive disorder. Increases in oxidative markers such as lipid peroxidase (LPO), TBARS, and MDA, along with decreases in antioxidant enzymatic systems (glutathione (GSH), catalase (CAT), superoxide dismutase (SOD), and glutathione peroxidase (GPX)), are observed in depressive disorder [117,118,119].

The AMY is one of the components of the limbic system responsible for controlling emotions and behavior, in addition to memory formation. The AMY plays a crucial role in regulating anxiety, aggression, stress responses, emotional memory, and social cognition [120,121]. Electrical stimulation of the AMY induces anxiety and fear responses in individuals, while lesions in this area inhibit certain types of unconditioned fear. Dysfunction of the AMY may lead to a lack of response to stimuli and, further, psychological disorders. Research has led many to conclude that hyperreactivity of the AMY increases the risk of depressive disorder [122]. Studies suggest that SGLT2 receptors are also found in the AMY [100]. However, no studies have conclusively demonstrated the modulation of specific molecular pathways within the AMY in established models of depression. However, Kamel et al. highlighted dapagliflozin’s (DAPA) anxiolytic effect in anxious demented rats. DAPA activated GABAB2 and promoted BDNF/TrkB and GABAA expression through the PLC/DAG/PKC pathway in an AMPK-dependent manner. Moreover, DAPA also restored levels of the AMY NE and 5-HT, along with suppressed glutamate levels [123].

**Table 2:** Summary of preclinical studies on SGLT2 inhibitors: effects on depressive-like behaviors and molecular pathways.

*Species/Strain*	*Model of Depression/Treatment*	*Results-Behavioral Tests*	*Samples*	*Methods*	*Results*	*References*
**Male Sprague–Dawley rats (6–8 weeks old)**	DM-induced depressive-like behavior; Dapagliflozin (1 mg/kg/day intragastric/500 ng/mL microinjection; *n* = 8)	↑ Total distance traveled, time spent in center area in the field test ↓ Immobility time in the forced swim test	Lateral habenula (LHb)	Western blot Immunostaining Chromatography	Intragastric administration: ↓ activity of LHb via ↓ c-Fos Microinjection into the LHb: ↑ 5-HT in the DRN Intragastric administration: ↑ p-AMPK, p-GABABR2 expression in the LHb	[124]
**Male Wistar rats (30–33 days old)**	Chronic unpredictable stress (CUS; 35 days); Dapagliflozin (1 mg/kg/day; 4 weeks; p.o.; *n* = 10)	↑ Sucrose consumption in the sucrose preference test ↓ Immobility time in the forced swim test	Hippocampus (HP) Serum Cortex	ELISA qRT-PCR Immunostaining	↑ 5-HT, DA, NE, BDNF in HP ↓ IL-1β, IL-18 in serum ↓ p-NF-κB p65, NLRP3, caspase-1 activity, IL-1β, IL-18 in HP ↓ ET-1, ↑ ET_B_R in HP ↑ BBB integrity: ↓ TNF-α in HP and cortex, ↑ ZO-1 cortex	[125]
**Male Wistar rats (weighing 180–200 g)**	Reserpine-induced depression (Res; 0.2 mg/kg/day; 14 days; i.p.); Escitalopram (10 mg/kg/day i.p.) Empagliflozin (10 mg/kg/day i.p.; *n* = 13)	↓ Immobility time in the tail suspension test and forced swim test ↑ Crossed squares in the open field test	Hippocampus (HP)	Western blot ELISA qRT-PCR Immunostaining	↑ 5-HT, DA, NE in HP ↑ p-AMPK, Beclin1, LC3B;↓ mTOR in HP ↑ GSH; ↓ MDA, NLRP3 caspase-1, IL-1β, IL-18, TNF-α in HP ↑ p-PKCζ, p-NF-kB p65, BDNF, p-CREB in HP	[126]
**Male Sprague Dawley rats (8 weeks old)**	Chronic unpredictable mild stress (CUMS; 28 days); Empagliflozin (10 mg/kg/day; 28 days; p.o.)	↓ Immobility time in the forced swim test ↓ Latency time; ↑ frequency of rearing, time and grooming in the open field test ↑ Grooming time in the splash test	Hippocampus (HP) Serum	ELISA Immunostaining	↑ 5-HT, NE, GSH in HP ↓ Serum corticosterone ↓ MDA, IL-1β, IL-18, NF-α, NF-κB, NLRP3, iba-1 in HP ↓ Hippocampal apoptosis (↓ cytochrome c)	[127]
**Male adult Wistar rats (weighing 150–200 g)**	Chronic unpredictable mild stress (CUMS; 7 weeks); Canagliflozin (20 mg/kg; 3 weeks)	↓ Latency time; ↑ frequency of grooming in the open field test ↓ Immobility time in the tail suspension test and forced swim test	Colon Serum Hippocampus (HP)	Western blot ELISA Immunostaining	↑Gut integrity (↑ E-cadherin, β-catenin, claudin-1, ZO-1, Goblet cells) ↓ Serum corticosterone ↓ SGLT2 receptors, IL-1β, IL-6, NF-κB, IDO in HP ↓ TNF-α, GFAP, IbA, CD86 in HP ↑ p-AMPK, Beclin-1 and LC3-II/I; ↓ p-mTor in HP	[128]
**Male adult BALB/C mice (weighing 24–30 g)**	Single-prolonged stress (SPS); Dapagliflozin (1 mg/kg/day; 7 days; oral gavage; *n* = 9)	↓ Immobility time in the tail suspension test and forced swim test	Prefrontal cortex (PFC) Serum	Real-time PCR ELISA	↓ Crh, IL-1β, BDNF, Bax mRna expression in PFC ↓ Serum corticosterone	[129]

Abbreviations: i.p.—intraperitoneally; p.o.—per-orally; (↑)—increase while; (↓)—decrease.

## 4. PPARα Agonists: Exploring the Neuroprotective and Metabolic Pathways

Recent advancements highlight the key role of disturbances in lipid metabolism in the pathogenesis and severity of depressive disorders [130]. Lipids, e.g., TGs, phospholipids, and cholesterol are vital in cell function. They constitute essential components of cell membranes, act as reservoirs for energy storage, and participate actively in signaling pathways. The metabolism of lipids is a fundamental biological process, involving their synthesis, degradation, and regulation within the organism, and underlies many essential physiological functions. The precise regulation of lipid metabolism enables the maintenance of cellular homeostasis and overall health [130]. Dyslipidemia, characterized by atypical blood lipid levels, is increasingly recognized as a key factor in the pathophysiology of depressive disorder. Extensive research has shown that changes in lipid profiles, especially higher TGs and lower HDL-C levels, correlate with an increased risk and greater severity of depressive disorder [13,14,131]. Dysregulation in lipid metabolism may impact neuronal function, leading to neuroinflammation, altered neurotransmission, oxidative stress, and disrupted neural plasticity, all of which are implicated in the development and progression of depressive symptoms. Furthermore, dyslipidemia may activate the HPA axis and trigger chronic low-grade inflammation, which has been associated with cognitive decline and mood disorders [66]. Anti-inflammatory agents such as omega-3 fatty acids have been demonstrated to alleviate these effects by promoting neurogenesis, enhancing synaptic plasticity, stabilizing membrane fluidity, and thereby reducing depressive symptoms [66].

PPARs are nuclear receptors that belong to the type II nuclear hormone receptor superfamily. They primarily regulate the transcription of genes involved in cell differentiation, proliferation, and apoptosis (both healthy and cancerous), as well as in lipid and glucose metabolism, energy homeostasis, the inflammatory response and oxidative stress. PPARα, PPARβ/δ, and PPARγ are three isoforms identified within the PPAR family [132]. Within the central nervous system (CNS), they are highly expressed and play a crucial role in regulating several neuronal processes, including the modulation of depressive behaviors. PPARα is the only detectable isotype in neurons, astrocytes, and microglia in the CNS of both rodents and humans. However, PPARα is also expressed in the PFC, AMY, basal ganglia, nucleus accumbens (NAc), ventral tegmental area (VTA), and thalamic nuclei. Its expression in the HP, however, is comparatively lower [133]. PPARα agonists primarily target lipid metabolism and are used to treat dyslipidemia and decrease triglyceride levels. Nevertheless, recent studies have shown an emerging role of PPARα and its agonist in the pathophysiology of depressive disorders [134]. Procedures inducing depressive-like behavior such as chronic social defeat stress, chronic unpredictable mild stress and chronic restraint stress significantly decreased PPARα expression in the hippocampus and enhanced immobility in the forced swim test [135,136]. Conversely, genetic hippocampal PPARα overexpression may induce significant antidepressant-like effects in mice by promoting CREB-mediated BDNF biosynthesis, while also reducing immobility time and restoring sucrose preference and social interaction to control levels [136]. Moreover, antidepressive drugs such as venlafaxine, fluoxetine and vortioxetine fully restored the decreasing effects of chronic stress protocols on hippocampal PPARα expression [135,136,137]. Matriscian et al. identified that the stress-induced epigenetic downregulation of PPARα in the HP is associated with hypermethylation of its promoter region [138]. PPARα agonists activate PPARα/RXR heterodimers, upregulating BDNF and suppressing NF-κB-mediated inflammation, thereby enhancing neuroplasticity. Fenofibrate is a synthetic PPARα agonist used in the treatment of dyslipidemia and may also exert antidepressant-like effects. Fenofibrate appears to exhibit these effects by restoring the chronic stress–induced decrease in the hippocampal BDNF signaling cascade and adult hippocampal neurogenesis [139]. Moreover, PPARα-associated antidepressant effects have also been linked to activation of the mesolimbic DA pathway. Fenofibrate restored sucrose preference and ventral tegmental area dopaminergic responses to appetitive stimuli in stressed rats, reversing behavioral deficits associated with anhedonia and impaired motivation, which are commonly observed in depressive disorder [140]. Gemfibrozil is an agonist of PPAR-α and may also have antidepressant effects [141,142]. Gemfibrozil fully reversed CUMS-induced depressive-like behaviors by significantly improving outcomes in the FST, TST, and sucrose preference test, while also restoring CUMS-induced inhibition of the hippocampal BDNF signaling pathway [141]. Furthermore, Zandifar et al. investigated the efficacy of gemfibrozil as a sertraline adjuvant in alleviating symptoms of MDD. Twenty-three patients were treated with gemfibrozil (300 mg/day) simultaneously with sertraline (100 mg/day) for 8 weeks. The combination treatment demonstrated promising results, with improvement in depressive symptoms assessed via HAM-D scores (achieved during patient follow-up at the second, fourth, and eighth weeks of the study) [142]. Bezafibrate, a PPARα agonist, is known as a lipid-lowering agent and is widely used in clinical therapeutics. Bezafibrate may significantly improve depression-like behaviors by increasing serum 5-HT levels, achieved through the promotion of the TRP-5-HT pathway and inhibition of the TRP-KYN pathway [143]. Studies have shown that another PPAR-α agonist, WY-14643, possesses antidepressive activities due to inhibiting neuroinflammation and oxidative stress. Pretreatment with WY-14643 suppressed the LPS-induced production of pro-inflammatory cytokines, such as IL-6, IL-1β, and TNF-α, and prevented the associated increase in oxidative and nitrosative stress in the PFC and HP [144]. Moreover, WY-14643 pretreatment reversed the LPS-induced decreases in hippocampal and prefrontal cortical BDNF levels. Additionally, WY-14643 increased levels of hippocampal BDNF in the CSDS-induced depressive mouse model [145]. The recent findings indicate that chiglitazar, a novel pan agonist of PPARs, exerts an antidepressive effect. Zhou et al. identified that the injection of chiglitazar significantly alleviated depressive-like behaviors in mice induced by CRS and CUMS in the FST, TST, and SPT. Furthermore, chiglitazar treatment completely restored the CUMS- and CRS-induced downregulation of hippocampal PPARα expression and BDNF signaling in mice [146].

## 5. Angiotensin Receptor Blockers and Angiotensin Receptor-Neprilysin Inhibitors and Their Impact on Depressive Disorders

Hypertension is highly prevalent in the general population and represents a key component of metabolic syndrome. There is a bidirectional relationship between human mood and HTN. Emotional and psychosocial stress may play a significant role in HTN development, with anxiety and depressive disorder negatively affecting blood pressure regulation. Conversely, patients diagnosed with HTN often experience emotional deterioration, including anxiety and depressive disorder, following diagnosis [18]. Multiple mechanisms are involved in the bidirectional association between depressive disorder and HTN. Chronic inflammation resulting from anxiety and depressive disorder contributes to the pathogenesis of HTN. The pathophysiological response is mediated by the dysregulation of the HPA axis, which is commonly observed in depressive disorders. Chronic activation of the HPA axis leads to elevated cortisol levels, promoting endothelial dysfunction, sympathetic nervous system overactivity, and increased vascular resistance, all of which contribute to the development of hypertension. Furthermore, decreased vagal tone, commonly observed in depressive disorder, is also thought to play a critical role in the pathophysiological response of HTN to stress [19]. Maladaptive behavioral responses, including a lack of physical activity, smoking, and poor dietary habits, are thought to contribute to the development of resistant HTN over time. Oxidative stress and inflammation play pivotal roles in the pathogenesis of HTN. Patients with HTN often exhibit elevated inflammatory biomarkers, such as CRP, and pro-inflammatory cytokines, including IL-6 and TNF-α. The bidirectional relationship between HTN and depressive disorders involves chronic inflammatory responses and pro-inflammatory factors, which are crucial in the pathogenesis of both conditions. Inflammation may interfere with nitric oxide (NO) bioavailability, impairing vasodilation and promoting vascular stiffness, while also contributing to neurotransmitter imbalance, particularly in serotonergic and dopaminergic pathways involved in mood regulation. Oxidative stress causes the increased production of reactive oxygen species (ROS) and reduced antioxidant capacity, leading to vascular damage and neuronal dysfunction [147]. A central feature is the rise in intracellular Ca^2+^ levels via increased Ca^2+^ influx through voltage-activated calcium channels (VACC). The sustained elevation of intracellular Ca^2+^ disrupts neuronal function and may result in cell loss in limbic brain structures, which are critical for emotional regulation. This may lead to dysregulation of the release of transmitters, e.g., 5-HT, causing depressive disorder. Concurrently, down-regulation of the cAMP signaling pathways (through decreasing the activity of Ca^2+^-sensitive ACs) results in an imbalance of the Ca^2+^/cAMP signaling interaction. This imbalance disrupts neurotransmitter release from sympathetic neurons, contributing to sympathetic hyperactivity and hypertension. Neuroinflammation, neurotransmitter imbalances, sympathetic overactivity, and interaction between altered Ca^2+^/cAMP signaling form a molecular basis for the frequent co-occurrence and mutual exacerbation of depressive disorder and hypertension [148].

The renin–angiotensin–aldosterone system (RAAS) is a hormone system that plays a pivotal role in regulating blood pressure, electrolyte and fluid balance, and systemic vascular resistance. RAAS is triggered by factors such as sympathetic nervous system activation, reduced sodium levels and low blood volume, leading to reduced renal perfusion. When activated, the RAAS stimulates the release of renin from the juxtaglomerular cells in the kidney. Renin converts a plasma protein called angiotensinogen, produced and released by the liver, into angiotensin I. Angiotensin I is subsequently cleaved by angiotensin-converting enzyme (ACE) activity to the active angiotensin II (Ang II). Ang II exerts complex hypertensive effects. It induces vasoconstriction via the type 1 angiotensin receptor (AT1R), which increases peripheral vascular resistance and elevates blood pressure. Additionally, activation of the AT1 receptor has pro-inflammatory effects, whereas AT2R mediates the effects of vasodilation and anti-inflammatory effects. Ang II also triggers the adrenal zona glomerulosa cells to release aldosterone, leading to sodium and water retention in the distal nephron, consequently increasing circulating blood volume [149]. Moreover, ang II augments the reflex activity of the sympathetic nervous system and stimulates the release of arginine vasopressin (AVP) from the posterior pituitary through the activation of the AT1 receptor within the brain. Increased RAAS activity is responsible for the hypertension. Chronic RAAS activation promotes vascular remodeling and endothelial dysfunction, sustaining hypertension and contributing to cardiovascular and renal complications [150].

In terms of the brain-specific localization of the RAAS, angiotensinogen, the main precursor of all angiotensin peptides, is produced predominantly (90%) by astrocytes, with minor contributions from neurons and glial cells in nearly all regions of the brain. Angiotensinogen must be cleaved by renin to form angiotensin peptides. Although renin is abundant in peripheral circulation, its brain levels appear at low concentrations. In contrast its precursor, pro-renin is present at higher concentrations and binds the pro-renin receptors with a higher affinity [151]. This interaction facilitates local angiotensinogen cleavage and the formation of angiotensin peptides. Angiotensin-converting enzymes ACE1 and ACE2 further cleave these peptides, with ACE1 mainly localized in the endothelia of cerebral vasculature and also in the choroid plexus, organum vasculosum of the lamina terminalis, subfornical organ and area postrema. Conversely, ACE2 is widely distributed, particularly in areas responsible for regulating central blood pressure [151]. Notably, increased Ang II levels are linked with depressive disorder, hyperactivity of the HPA axis, neuroinflammation, and stress. Park et al. indicate that the chronic infusion of Ang II into mice induces depressive-like behaviors, including the TST and FST. Moreover, it also induces activation of the microglia in the HP with an increase in Il-1β mRNA and a decrease in Arg1 mRNA, and pro-inflammatory changes in BV-2 microglial cells. Additionally, chronic Ang II infusion activated the HPA axis, resulting in decreased levels of the hippocampal glucocorticoid receptor [152]. On the other hand, ACE2 metabolizes Ang II to Ang-(1-7), which is essential for the intestinal uptake of 5-HT precursor, i.e., TRY. Ang-(1-7) plays a neuroprotective role and antagonizes Ang II, exerting antidepressant effects via the Mas receptor (MasR) [153]. Depressive disorder may exhibit decreased expression levels of ACE2 and Ang (1-7), and the depletion of ACE2 may further decrease brain 5-HT levels [154]. Recent studies have shown that both oral and intranasal Ang-(1-7) treatments effectively alleviate depressive and anxiety-like behaviors, by decreasing TNFα and IL-6 levels in the PFC and increasing IL-10 levels [155]. Angiotensin receptor blockers (ARBs) are a class of medications that selectively inhibit the binding of angiotensin II to the angiotensin type. ARBs may increase the circulating levels of angiotensin (1-7), but unfortunately, no studies have specifically investigated the effects of ARB treatment of Ang-(1-7) in animal models of depression [156].

In the CNS, angiotensin exerts its effects particularly via two receptor subtypes, AT1 and AT2. The central AT1 receptor (AT1R) exhibits dense expression in subcortical, limbic, and frontal brain regions, such as the thalamus, striatum, AMY, HP, and PFC, and may therefore serve as an important target for regulating cognitive and affective disturbances associated with depressive disorders. Investigation of the AT1R gene expression map in the brain through behavioral decoding associations with stress, memory, reward, and motivational processes [157]. Cognitive and affective domains such as memory, stress regulation, reward processing, and motivational control are central to the pathophysiology of depressive disorders. Memory dysfunction in depressive disorder has been linked to hippocampal impairment and reduced synaptic plasticity as a consequence of chronic stress and HPA axis overactivation. Exposure to stress, observed in depressive disorder, is associated with a reduced ability to experience pleasure. Anhedonia reflects impaired reward processing and motivation, which are associated with a dysregulated mesocorticolimbic DA system [158]. The pharmacological blockade of AT1R with losartan decreased neural activity in subcortical systems while increasing functional connectivity within the cortico–basal ganglia–thalamo–cortical circuitry [157]. Alterations in this circuit play a crucial role in the pathophysiology of MDD [159]. The effects of AT1R blockade on the network level were specifically associated with the transcriptomic signatures of the dopaminergic, opioid, acetylcholine, and corticotropin-releasing hormone signaling systems—neurotransmitter pathways known to be dysregulated in depressive disorder [157]. Moreover, valsartan treatment significantly improved oxidative stress parameters (increased GSH, decreased MDA levels) and increased BDNF levels in the menopause-induced depression mouse model [160]. Increased BBB permeability is increasingly recognized as a factor in depressive disorder [161]. The MLCK/MLC signaling pathway regulates brain vascular endothelial cell permeability. The phosphorylation of MLC by MLCK may promote increased BBB permeability [162]. Irbesartan may reduce the permeability of the BBB by restoring the expression of Occludin and mediating the NF-κB/MLC/MLCK signaling pathway; notably, NF-κB inhibition abolished Irbesartan’s effects. In addition, irbesartan may suppress the expression of inflammatory mediators, including IL-6, intercellular adhesion molecule-1 (ICAM-1) and monocyte chemoattractant protein-1 (MCP-1) [163]. Gouveia et al. demonstrated that ARBs, particularly irbesartan, exhibited more potent anti-inflammatory effects than ACE inhibitors in BV-2 microglial cells, significantly reducing iNOS and pro-IL1β protein levels and nitrite production. In an LPS-induced mouse model, the intranasal administration of irbesartan reversed cognitive impairment and depressive-like behavior, restored PI3K/AKT signaling, increased p-GSK3β levels, enhanced antioxidant defenses by upregulating SOD2 protein and Gpx1 mRNA levels, and improved markers of neuronal survival and synaptic plasticity (CREB1, BDNF) in the HP. Moreover, irbesartan reduced hippocampal astrogliosis and preserved dendritic spine density in PFC pyramidal neurons, indicating neuroprotective and mood-enhancing effects [164]. Irbesartan may also exert an antidepressant-like effect by elevating brain 5-HT levels [165]. TrkB is a tyrosine kinase receptor that is activated upon binding to BDNF, and the localization of TrkB receptors is determined by the tyrosine kinase Fyn [166]. The antidepressant-like effect of losartan enhances Ang II binding to AT2R by inhibiting AT1R, leading to increased TrkB levels and the coupling of TRK/FYN in the hippocampus and ventromedial PFC (vmPFC) prelimbic regions [167]. Notably, an extensive cohort study demonstrates that ARBs are associated with the lowest rates of affective, anxiety, and psychotic disorders compared to other antihypertensive drug classes [168].

Angiotensin receptor-neprilysin inhibitors (ARNIs) represent a novel class of cardiovascular therapeutics characterized by their action on the two key regulators of the cardiovascular system, including RAS and the natriuretic peptide (NP) system [169]. Recent studies show that sacubitril/valsartan, an angiotensin receptor-neprilysin inhibitor, may decrease anxiety and depression symptoms in patients with heart failure with a reduced ejection fraction (HFrEF) [170]. Surprisingly, in patients with HFrEF, the switch from ACEI/ARB treatment to sacubitril/valsartan resulted in a significant improvement in depressive disorder, anxiety symptoms, and functional statuses [171]. Additionally, sacubitril/valsartan therapy was also linked to sustained medium-term quality of life (QOL) improvements in cognitive function, sleep, social functioning and daily activities in patients with systemic right ventricle failure [172]. Unfortunately, no preclinical studies that investigated the antidepressive effects of ARNIs in animal models of depression have been identified.

## 6. Concluding Remarks and Future Perspectives

An increasing number of studies have confirmed that depressive disorder is comorbid with a variety of metabolic diseases, such as diabetes, obesity, dyslipidemia or hypertension. Metabolic diseases and depressive disorder may have a complex bidirectional relationship underpinned by overlapping biological and psychosocial pathways. The emerging evidence linking metabolic dysregulation and the pathophysiology of depressive disorder opens novel therapeutic targets beyond monoaminergic approaches, which often have limited effectiveness. Metabolic regulators and cardiovascular agents, including GLP-1 agonists, SGLT2 Inhibitors, PPARα agonists, and modulators of the RAA system, namely ARBs and ARNIs, have demonstrated promising results in preclinical studies and, in some instances, in early clinical observations, improving depressive-like behaviors. As previously described, GLP-1 RAs mitigate depression via enhancing central insulin signaling, restoring monoamine balance (5-HT, DA, NE), and attenuating oxidative stress and neuroinflammation by downregulating the expression of GSDMD, TBARS, Iba1, IL-1β, IL-6, TNF-α, and NF-κB p65, among others. Furthermore, they promote hippocampal neurogenesis and synaptic plasticity by activating the PI3K/AKT, CREB, and BDNF pathways, as well as modulating neuronal lipid metabolism (e.g., NAA). Additionally, GLP-1RAs decrease plasma ACTH and serum CORT and CRP levels, thereby reducing HPA axis hyperactivity and systemic inflammation (Figure 1, schematic of GLP-1R–mediated antidepressant mechanisms) (Table 1, preclinical studies summary). Similarly, SGLT2 inhibitors alleviate depressive-like behaviors by attenuating oxidative stress, neuroinflammation through decreasing p-NF-κB p65, IL-1β, IL-18, TNF-α, caspase-1 activity, and NLRP3 inflammasome activation. They also restore BBB integrity (↓ET-1, ↑ ETBR in HP and ↑ ZO-1 in cortex) and regulate TRY-KYN metabolism by decreasing the acitivity of IDO. Moreover, SGLT2 inhibitors enhance autophagy via AMPK/mTOR signaling, promote neurogenesis and modulate neurotransmitter systems in key mood-related regions such as the LHb and HP (Figure 2, schematic of SGLT2–mediated antidepressant mechanisms), (Table 2, preclinical studies summary). PPARα agonists improve hippocampal BDNF signaling and neurogenesis, modulate CREB-dependent transcription and reduce neuroinflammation and oxidative stress through the inhibition of pro-inflammatory cytokines (IL-6, IL-1β, TNF-α). Additionally, they restore mesolimbic DA pathway activation, regulate TRP–5-HT/KYN metabolism and improve depressive symptoms in both preclinical and clinical studies (assessed via HAM-D scores). Finally, ARBs exert antidepressive effects by blocking AT1R signaling, thereby reducing neuroinflammation (IL-6, ICAM-1, MCP-1, iNOS and pro-IL1β), oxidative stress (increased GSH, decreased MDA levels) and BBB permeability (restoring the expression of Occludin mediating via the NF-κB/MLC/MLCK signaling pathway) while enhancing BDNF/CREB and TrkB signaling. However, the antidepressant efficacy of ARNIs has only been demonstrated in clinical studies that showed significant improvements in both depressive disorder and anxiety symptoms. These findings suggest that targeting peripheral metabolic pathways and their interactions with the central nervous system may alleviate depressive symptoms through multiple mechanisms, including improvements in neuroplasticity and neurogenesis, the amelioration of oxidative stress and neuroinflammation, the regulation of neurotransmitter levels, the HPA axis, and mitochondrial function.

Despite these encouraging results, substantial gaps remain in these findings. The majority of available data derive from animal studies or secondary analyses of cardiovascular or diabetes trials, with limited randomized clinical trials specifically designed to evaluate antidepressant efficacy. Notably, there is a lack of studies on the effects of these agents on the modulation of molecular pathways implicated in depressive disorder, particularly human-derived samples, such as postmortem brain tissue. Additionally, future research should also focus on assessing the effects of these drugs on molecular pathways within brain regions other than the hippocampus, which are known to be involved in depression (e.g., thalamus, AMY, frontal cortex, PFC). Most preclinical studies focus on Hp as the primary region of investigation; however, this structure is not the only CNS area implicated in the pathophysiology of depressive disorder. For instance, within preclinical research on SGLT2 inhibitors, no studies to date have examined the AMY, despite its well-established role in mood regulation. Moreover, future studies should use continuous glucose monitoring to isolate metabolic effects from glycemic variability. Furthermore, current clinical trials evaluating GLP-1 receptor agonists and SGLT2 inhibitors in the context of depressive disorders are limited by relatively short follow-up periods, emphasizing the need for more extended follow-up periods to assess their neuropsychiatric potential fully. This limitation likely reflects the relatively recent introduction of these therapeutic classes into clinical practice. A multicenter, double-blind, placebo-controlled randomized controlled trial (*n* ≥ 700) is warranted in patients with comorbid depressive disorder and diabetes, employing both HAM-D scores and HbA1c levels as dual endpoints over extended follow-up periods (≥12 months). Moreover, there is a lack of studies evaluating the dose-dependent efficacy of these agents in reducing depressive symptoms. Importantly, current clinical evidence regarding the impact of GLP-1 analogs on depressive symptoms remains contradictory. While some trials support their effectiveness in alleviating depressive symptoms, others have reported contrary findings, with indications that GLP-1 analogs may exacerbate mood disturbances and, in rare cases, contribute to suicide attempts. In addition, given that all the reviewed drug classes exhibit antidepressant effects, future research should focus on comparative evaluations or investigate their role as adjunctive therapy to conventional agents to determine their relative efficacy and optimize therapeutic selection. Similarly, no clinical investigations have systematically compared outcomes across more than two patient subgroups, for example, by assessing whether GLP-1 agonists demonstrate equivalent efficacy in individuals with T2DM, obesity, combined T2DM and obesity, or T2DM/obesity with additional comorbidities—despite the likelihood that treatment responses differ across these populations. Furthermore, there is a lack of head-to-head comparisons of individual GLP-1 analogs, even though available clinical data suggest that semaglutide, liraglutide, and dulaglutide may exert differential effects on the alleviation or exacerbation of depressive symptoms. A recent in silico analysis showed that the GLP-1 receptor agonist semaglutide interacts with dopaminergic and neurotrophic signaling pathways (DRD3, BDNF, CREB1, CRH, IL-6, DPP4), which are critically implicated in depressive disorder and mood regulation. While GLP1R agonism may be beneficial in the treatment of hyperdopaminergia, in conditions of hypodopaminergia it may be detrimental, potentially leading to the exacerbation of depressive phenotypes and the long-term induction of suicidal ideation. In individuals carrying reward gene polymorphisms indicative of reduced dopaminergic function, as assessed by the Genetic Addiction Risk Score (GARS), the long-term administration of GLP-1 agonists should therefore be approached with caution. In such patients, dosage adjustment or therapeutic substitution may be required to prevent the further downregulation of DA release in the NAc. This study partially explains the inconsistent findings of a clinical trial investigating the effects of GLP-1 analogs on depressive symptoms. Collectively, these findings highlight the dual pharmacometabolic effects of GLP-1 receptor agonism and emphasize further clinical validation [173]. From a biomarker perspective, the in silico analysis highlights several candidate molecular signatures that may bridge GLP-1R signaling with depressive phenotypes. Markers such as DRD3, BDNF, CREB1, CRH, IL6, and DPP4 emerge as relevant targets reflecting neuroimmune-metabolic interactions. In parallel, circulating microRNAs—including hsa-miR-22-3p, hsa-miR-16, hsa-miR-22, hsa-miR-195 and hsa-miR-204-5p—were identified as central regulatory hubs and may serve as accessible plasma biomarkers for monitoring susceptibility to depressive symptoms or suicidal ideation during GLP-1RA treatment. Integrating these signals with routine metabolic measures such as HbA1c, indices of insulin resistance and lipid profiles may ultimately enable a pharmacometabolomic framework linking systemic metabolic modulation to neuropsychiatric outcomes in patients under GLP-1RAs therapy. Future research should prioritize the empirical validation of the associations identified in the in silico analysis through well-designed clinical studies. Moreover, comparable in silico analysis are lacking for other GLP-1 receptor agonists as well as for SGLT2 inhibitors.

In this review, we focused only on selected classes of metabolic drugs, primarily GLP-1 receptor agonists and SGLT2 inhibitors, due to the relatively high number of preclinical studies assessing their impact on depressive-like behaviors in animal models of depression. We also discussed PPARα agonists and RAA modulators such as ARBs and ARNIs, although preclinical and clinical data on these drug classes remain limited. However, an increasing number of preclinical and clinical studies highlight the potential antidepressant effects of other metabolic drug classes, such as dipeptidyl peptidase-4 (DPP-4) inhibitors, proprotein convertase subtilisin/kexin type 9 (PCSK9) inhibitors, and angiotensin-converting enzyme inhibitors (ACEIs), which are not included in this review. Metabolic modulators show promise as an adjunctive therapy in depressed patients with comorbid metabolic syndromes. These agents represent promising avenues for future research, especially given the rising prevalence of both depressive disorder and metabolic disorders, and the bidirectional relationship between these conditions. Furthermore, the growing number of individuals suffering from treatment-resistant depression underscores the need to explore alternative therapeutic strategies that extend beyond the monoamine hypothesis. Such a strategy may target other molecular pathways, including HPA axis dysregulation, cytokine signaling disturbances, mitochondrial dysfunction, oxidative stress, neuroinflammation, and impaired neuroplasticity—mechanisms increasingly recognized as shared pathophysiological features of both depressive disorder and metabolic disorders.

## 7. Limitations

Growing evidence highlights the role of metabolic modulators in depressive disorder; however, this field remains constrained by several important limitations that must be acknowledged. One key limitation is the lack of an evaluation of dose–response relationships, as most preclinical studies employed a single fixed dose rather than multiple dosing regimens, thereby limiting conclusions about the dose dependency of the observed antidepressant effects. Additionally, in terms of effect size analysis, effect sizes could not be reliably calculated, as most studies reported only group-level results, without the raw data necessary for standardized measures (e.g., Cohen’s d). Moreover, a further limitation is the inability to evaluate long-term safety, as preclinical studies are rarely conducted under long-term conditions.

## Data Availability

This review article relies exclusively on previously published data, fully cited in the bibliography. The original datasets used in the cited studies were not accessed or analyzed by the authors.
